# Itk Derived Signals Regulate the Expression of Th-POK and Controls the Development of CD4^+^ T Cells

**DOI:** 10.1371/journal.pone.0008891

**Published:** 2010-01-26

**Authors:** Jianfang Hu, Qian Qi, Avery August

**Affiliations:** 1 Center for Molecular Immunology and Infectious Disease, Department of Veterinary and Biomedical Sciences, The Pennsylvania State University, University Park, Pennsylvania, United States of America; 2 Immunology and Infectious Disease Graduate Program, The Pennsylvania State University, University Park, Pennsylvania, United States of America; New York University, United States of America

## Abstract

T cell development is critically dependent on both the environment and signals delivered by the T cell Receptor (TCR). The Tec family kinase Itk has been suggested to be an amplifier of signals emanating from the TCR and the loss of Itk partially affects most stages of thymopoiesis. Loss of Itk also differentially affects the development of conventional vs. non-conventional or innate memory phenotype T cells. Here, we examine whether these lineage choices are affected by a combination of TCR affinity and Itk by analyzing mice lacking Itk and carrying two TCR transgenes with differing affinities, OT-II and DO11.10. Our results show that developing thymocytes receive a gradient of signals, DO11.10>OT-II>DO11.10/Itk^−/−^>OT-II/Itk^−/−^. We also show that the development of CD4^+^ T cells is controlled by TCR signaling via Itk, which regulates the expression of the transcription factor, Th-POK, an enforcement factor for CD4 commitment. This results in a reduction in CD4^+^ T cell development, and an increase in the development of MHC class II restricted TCR transgenic CD8^+^ T cells that resemble non-conventional or innate memory phenotype CD8 T cells. This alteration accompanies increased expression of Runx3 and its target genes Eomesodermin, Granzyme B and Perforin in Itk null OT-II CD4^+^ thymocytes. All together, these data suggest that Itk plays an important role in CD4/CD8 commitment by regulating signal thresholds for the lineage commitment. Our data also suggest that the lower level of TCR signaling that occurs with a low affinity TCR in the absence of Itk can redirect some MHC class II restricted CD4^+^ T cell to class II-restricted CD8^+^ innate memory phenotype T cells.

## Introduction

Mature T cell development takes place in the thymus and is critically dependent on signals through the TCR. During T cell development, TCR signals generated by interaction with major histocompatibility complex class (MHC)-II peptide complexes are required for differentiation of CD4^+^ T cells, while TCR signals generated by interaction with class I MHC-peptide complexes are required for differentiation of CD8^+^ T cells. This process, referred as CD4 and CD8 commitment, is a major developmental process after positive and negative selection. While TCR signals are important for this lineage, they are not completely clarified [1], [2], [3]. High signaling activity generated by the tyrosine kinase Lck or MAP kinases ERK1/2 enhances CD4 Single Positive (SP) development [4], [5], while low activity of Lck, ZAP70 or ERK1/2 leads to CD8 SP development [4], [5], [6]. These findings support the idea that attenuating TCR signaling could redirect thymocytes with class II restricted TCRs from CD4 to the CD8 lineage, while enhanced signaling could redirect thymocytes with class I specific TCRs from CD8 to CD4 lineage. Other evidence indicates that the duration of signaling as well as the number of TCRs triggered are key factors in determining CD4/CD8 T cell fate decision [7], [8]. A role for TCR affinity has not been implicated in these events, but may play a role dependent on whether TCR affinity affects these implicated signaling events.

The IL-2-inducible T cell kinase (Itk), a Tec family kinase, regulates TCR signals and is considered an amplifier of these signals in T cells [9], [10], [11], [12]. Itk is directly activated by Lck and in turn plays a role in the activation of PLCγ1, Ca^2+^mobilization and activation of ERK/MAPK [13]. Itk-deficient mice had decreased numbers of mature thymocytes [14], [15], [16], and exhibit altered thymopoiesis, including pre-TCR signaling, as well as positive and negative selection [15], [17], [18], [19]. Based on these observations and the important roles of other TCR signaling proteins such as Lck and ZAP70 in the CD4/CD8 commitment, it seems likely that the Itk may also influence CD4/CD8 commitment [20]. However, Berg and colleagues have examined this issue by crossing Itk deficient mice to mice carrying the AND MHC class II restricted TCR, along with different MHCs that had varying affinities for TCR, and observed that while Itk affects positive selection and development of CD4 SP thymocytes, CD8 SP thymocyte development is not affected, suggesting that the Itk signaling is not involved in the lineage commitment [18]. However, these experiments did not rule out the possibility that signals in the TCR transgenic systems used in this study may be above a certain threshold that, even in the absence of Itk, does not affect CD4 and CD8 lineage commitment.

We and others have recently reported that large majority of CD8^+^ T cells in mice lacking Itk have the characteristics of memory cells and innate immune cells [21], [22], [23], [24], [25]. These cells have been referred to as “non-conventional CD8^+^ T cells” or “innate memory phenotype cells”. By contrast, cells carrying the surface phenotype of naïve CD8^+^ T cells have the characteristics of conventional CD8^+^ T cells, and are drastically reduced in the absence of Itk. These data suggest that Itk modulated signals may regulate the development of specific subpopulations of CD8^+^ T cells. However, it is not clear if these CD8^+^ T cells develop because they are destined to become CD8^+^ T cells, and the Itk signals regulate whether they develop into conventional or non-conventional CD8^+^ T cells, or whether Itk signals regulate the development of these cells regardless of their initial cell fate choice. Here, we have analyzed mice lacking Itk and carrying two low affinity TCR transgenes specific for a peptide derived from Ovalbumin (OT-II mice, DO11.10, presented by different respective MHC class II molecules in C57BL/6 and BALB/c mice), to determine if cells destined to become class II restricted CD4^+^ T cells are affected by the absence of Itk [26]. Our results show that compared to thymocytes developing in DO11.10/Itk^−/−^ mice, thymocytes in OT-II/Itk^−/−^ mice receive reduced TCR signals, with reduced development of “naïve” transgenic CD4^+^ T cells, and subsequent development of significant numbers of peptide specific MHC class II restricted TCR transgenic CD8^+^ T cells. Furthermore, a large majority of these TCR transgenic CD8^+^ T cells have the characteristics of non-conventional or innate memory phenotype CD8^+^ T cells. We also show that the absence of Itk affects the expression of Th-POK (T help inducing POK factor), Runx3 (runt-related transcription factor 3) and TOX (thymocyte selection-associated HMG box), transcription factors that regulate the development of CD4 and CD8^+^ T cells. These data indicate that the absence of Itk allows the development of non-conventional CD8^+^ T cells even if they carry a class II restricted TCR, and suggest that the development of these cells is regulated in part by altered signals from this low affinity TCR. Altered development of CD4^+^ naïve T cells in the absence of Itk may be a consequence of reduced TCR signaling, resulting in reduced expression of the transcription factor, Th-POK, a master regulator of CD4 commitment, and increased expression of Runx3 with accompanying changes in cell fate decision.

## Results

### The Absence of Itk Results in Reduced Development of CD4^+^ T Cells

**Figure 1:**
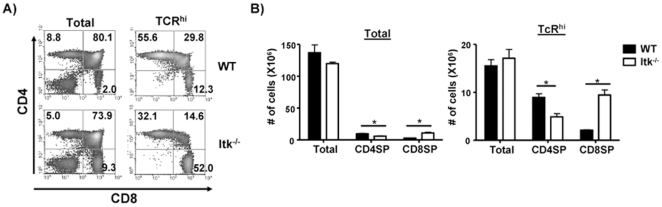
The absence of Itk affects the development of CD4 and CD8 T cells. (**A**) Thymocytes from WT or Itk^−/−^ mice were analyzed for expression of CD4 and CD8 (left), or gated first on the TCR^hi^ population and analyzed for CD4 and CD8 (right). (**B**) The number of thymocytes (total, left) or TCR^hi^ thymocytes (right) in WT and Itk^−/−^ mice. *p<0.05, n = 3–5 mice.

We compared the development of CD4 and CD8 single-positive (SP) T cells in the thymi of mice lacking Itk and found, as previously reported, that the absence of Itk results in reduced percentages of CD4 SP T cells, with an increase in the percentage of CD8 SP T cells (**Fig. 1a**) [27], [28], [29], [30], [31], [32]. Gating on the more mature TCR^hi^ cells illustrates this point more keenly, with a significant reduction in TCR^hi^ CD4 SP cells in mice lacking Itk, and an increase in TCR^hi^ CD8 SP cells. As we and others have previously reported, this alteration in the percentages of CD4 and CD8 SP cells reflects reduced numbers of TCR^hi^ CD4 single positive cells, with an increase in TCR^hi^ CD8 SP cells (**Fig. 1b**).

**Figure 2:**
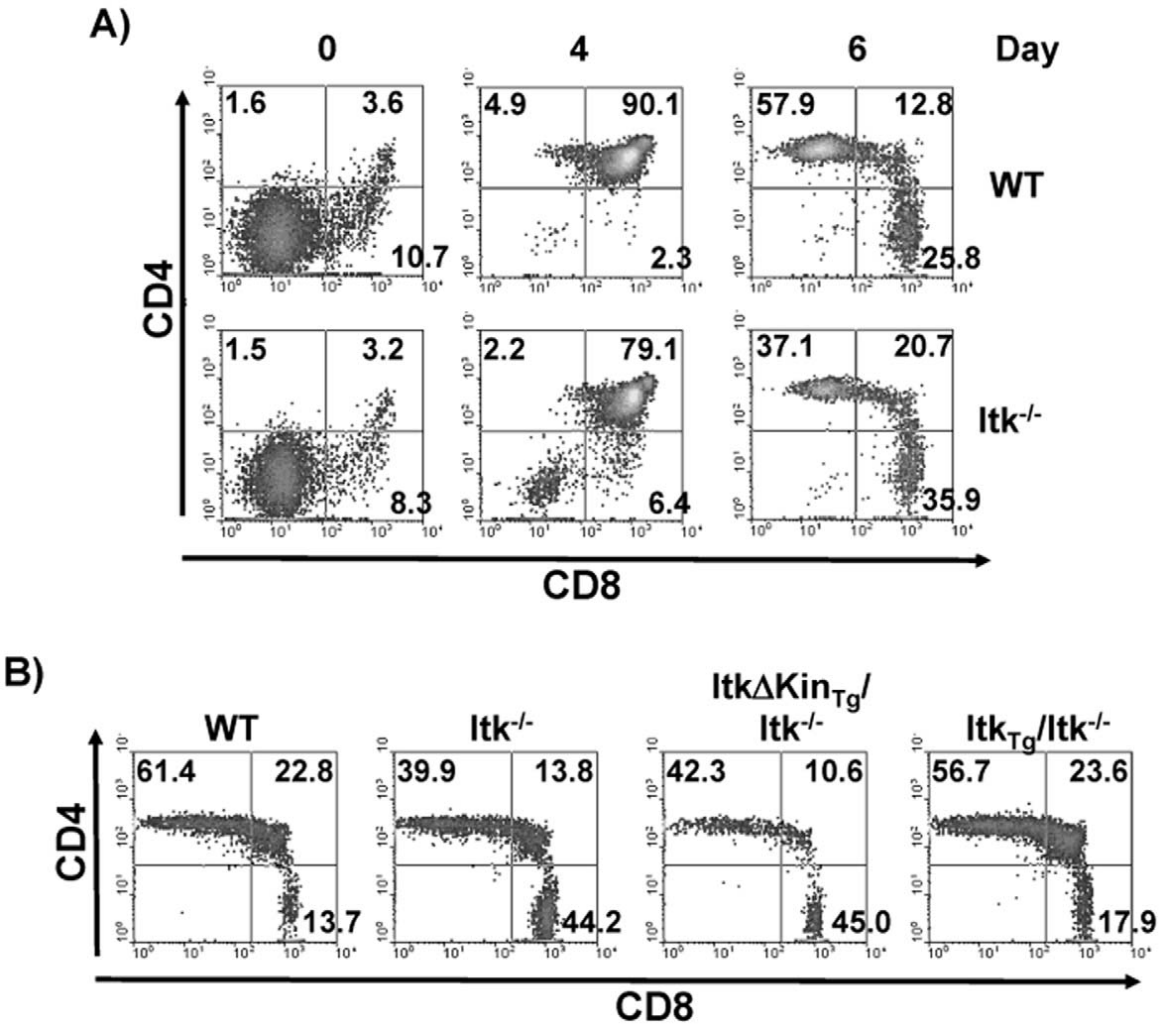
(A) Fetal Thymic Organ Culture analysis of WT and Itk^−/−^ thymocyte development. FTOC from embryonic day 16 of WT and Itk null mice incubated in vitro for the indicated days, gated on TCR^hi^ and analyzed for CD4 and CD8 (data representative of 3–4 mice). **(B) Active Itk kinase activity is required for T cell development.** Thymocytes from WT, Itk^−/−^, *Tg(Lck-ItkΔKin)Itk^−/−^* and *Tg(CD2-Itk_tg_)Itk^−/−^* mice were gated first on the TCR^hi^ and analyzed for expression of CD4 and CD8.

We next performed fetal thymic organ cultures to determine if this alteration in development is due to alterations in the timing of development of these cells. Figure 2 demonstrates that compared to WT thymocytes, those lacking Itk exhibited reduced development of CD4SP T cells in vitro, with increased percentage of CD8 SP T cells developing (**Fig. 2a**). These data demonstrate that the absence of Itk affects the development of both CD4^+^ and CD8^+^ T cells.

Further analysis of transgenic mice carrying WT or a kinase domain deleted mutant of Itk in a T cell specific fashion illustrates that kinase activity of Itk is required for efficient development of these T cells. Gating on mature TCR^hi^ thymocytes we compared Itk null mice carrying a WT Itk transgene (*Tg(CD2-Itk_tg_)Itk^−/−^)* to WT mice, and found that WT Itk was able to rescue the ratio of CD4 to CD8 T cells, particularly in the TCR^hi^ SP population (**Fig. 2b**). By contrast, the previously described kinase domain deleted mutant Itk (*Tg(Lck-ItkΔKin)Itk^−/−^*) [27], was unable to rescue the ratio of CD4 to CD8 T cells. In the experiments detailed below, we further characterize the role of Itk in the development of CD4^+^ T cells, and return to CD8^+^ T cells later in this report.

### The Absence of Itk Results in Reduced Development of OT-II Transgenic CD4^+^ T Cells

**Figure 3:**
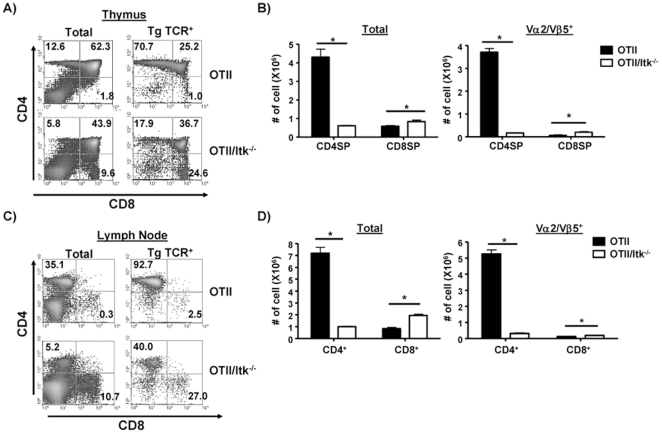
The absence of Itk affects the development of CD4^+^ T cells in OT-11 transgenic mice. (**A**) Total thymocytes (left panels) or the transgenic TCR^+^ (Vα2/Vβ5) population from OT-II and OT-II/Itk^−/−^ mice analyzed for expression of CD4 and CD8 (right panel). (**B**) The number of thymocytes (total, left) or transgene TCR^hi^ thymocytes (right) in WT and Itk^−/−^ mice. *p<0.05, n = 3–5 mice. (**C**) Total lymphocytes (left panel) or the transgenic TCR^+^ (Vα2/Vβ5) population (right panel) from cervical lymph nodes of OT-II and OT-II/Itk^−/−^ mice analyzed for expression of CD4 and CD8. (**D**) The number of total lymphocytes (left) or the transgene TCR^+^ T cells from cervical lymph nodes (n = 3–5, *p<0.05).

Previous analysis of the role of Itk in T cell development using TCR transgenes that drive CD4^+^ T cell development have not revealed a role in CD4 or CD8 lineage commitment [15], [16]. However, these transgenes may have had affinities for antigen that were high enough to overcome any differences. Therefore to determine if reduced TCR signals due to the absence of Itk can influence CD4 lineage development, we crossed Itk^−/−^ mice to TCR transgenic OT-II mice. OT-II mice carry a transgenic αβ TCR (Vα2/Vβ5) that recognizes ovalbumin 323–339 in the context of MHC class II I-A^b^ with low affinity [26], [33]. Greater than 95% of TCR transgene positive T cells are CD4^+^ T cells (**Fig. 3a** **,** see **figure S1** for TCR transgene expression profiles). However, the absence of Itk in OT-II mice dramatically reduced the development of CD4 SP cells, accompanied by the development of a significant percentage of CD8 SP cells that were positive for the TCR transgene TCR positive (**Fig. 3a**). This resulted in a ratio of CD4∶CD8 TCR transgene positive cells of 0.7 in OT-II/Itk^−/−^ mice, compared to 70 for the WT OT-II mice, a change of greater than 100 fold.

In the lymph node, the ratio of CD4∶CD8 TCR transgenic T cells was 37 in the WT OT-II mice compared to 1.5 in the OT-II/Itk^−/−^ mice, a change of 25 fold (**Fig. 3c**). These data suggest that Itk is essential for the development of OT-II thymocytes into CD4 lineage cells. Our data also suggest that in the absence of Itk, developing double positive (DP) thymocytes may have a slight preference for becoming CD8 SP cells, a point we will discuss further later.

Although the OT-II/Itk^−/−^ mice showed a 3–4 fold reduction in total thymocytes that were TCR transgene positive compared to OT-II mice (data not shown), the number of TCR transgene positive CD8 SP thymocytes was increased 2–3 fold, while that of CD4 SP thymocytes was reduced 5–6 fold (**Fig. 3b**). This difference between WT and Itk^−/−^ TCR transgene positive CD4 SP cells was more exaggerated in the lymph node, where we observed a 15–16 fold reduction (**Fig. 3d**). This could be the result of reduced homeostatic expansion of transgene positive CD4^+^ T cells in the absence of Itk after migrating into periphery, as this was observed in the non-transgenic mice when naïve T cells from Itk^−/−^ mice were transferred into RAG^−/−^ mice (data not shown). In addition, the increase in TCR transgene positive CD8^+^ T cells observed in Itk^−/−^ mice was less pronounced in the lymph node, with a ∼1.5 increase compared to WT OT-II mice (**Fig. 3d** **,** similar results were seen in the spleen, data not shown).

### The Absence of Itk Has Less of an Effect on the Development of DO11.10 Transgenic CD4^+^ T Cells

We next asked whether the affinity of the TCR was important for our finding of a significant role for Itk in CD4^+^ T cell development. The OT-II transgenic TCR is a low affinity TCR, and recognizes the same antigen as the transgenic TCR carried by DO11.10 transgenic mice, albeit presented by a different MHC class II molecule [26]. The DO11.10 TCR carries the transgenic αβ TCR (Vα13/Vβ8) that also recognizes ovalbumin 323–339, however, in the context of MHC class II I-A^d^ and has approximately 50 fold higher affinity for antigen than OT-II [26], [33]. We therefore determined if the absence of Itk on the DO11.10 transgenic background would have less of an effect on CD4^+^ and CD8^+^ T cell development.

**Figure 4:**
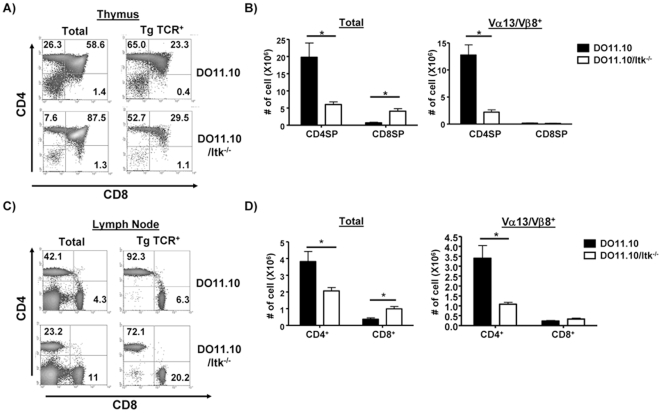
The absence of Itk affects the development of CD4^+^ T cells in DO11.10 transgenic mice. (**A**) Total thymocytes (left panels) or the transgenic TCR^+^ (KJ-11^+^) population from DO11.10 and DO11.10/Itk^−/−^ mice analyzed for expression of CD4 and CD8 (right panel). (**B**) The number of thymocytes (total, left) or transgene TCR^hi^ thymocytes (right) in WT and Itk^−/−^ mice. *p<0.05, n = 3–5 mice. (**C**) Total lymphocytes (left panel) or the transgenic TCR^+^ population (right panel) from cervical lymph nodes of DO11.10 and DO11.10/Itk^−/−^ mice analyzed for expression of CD4 and CD8. (D) The number of total lymphocytes (left) or the transgene TCR^+^ T cells from cervical lymph nodes (n = 3–5, *p<0.05).

To this end, we analyzed Itk^−/−^ mice crossed to TCR transgenic DO11.10 mice. Supporting the view that the affinity of the TCR is critical for the function of Itk in the development of CD4^+^ T cells, the absence of Itk had less of an effect on the development of CD4 SP cells in DO11.10 mice with the higher affinity TCR (**Fig. 4a** **,** see **figure S1** for TCR transgene expression profiles). This resulted in a ratio of CD4∶CD8 TCR transgene positive of ∼162 in WT DO11.10 mice, compared to ∼50 for the DO11.10/Itk^−/−^ mice, a change of ∼3 fold. This was also reflected in the numbers of these cells (∼4 fold difference in CD4SP cell number, **Fig. 4b**).

In the lymph nodes of the DO11.10 mice, the TCR transgene positive CD4∶CD8 ratio was ∼14.7 compared to ∼3.6 in the DO11.10/Itk^−/−^ mice, a change of ∼4 fold (**Fig. 4c**). This was also reflected in the numbers of these cells (∼3.5 fold difference in CD4SP cell number, **Fig. 4d**). These data suggest that the affinity of the TCR is critical in determining whether Itk will be important for the development of CD4^+^ thymocytes, and that a high affinity TCR can partially or fully compensate for the lack of Itk in CD4^+^ thymocyte development.

### Interaction between TCR Affinity and Itk Derived Signals Regulate the Development of CD4^+^ T Cells

**Figure 5:**
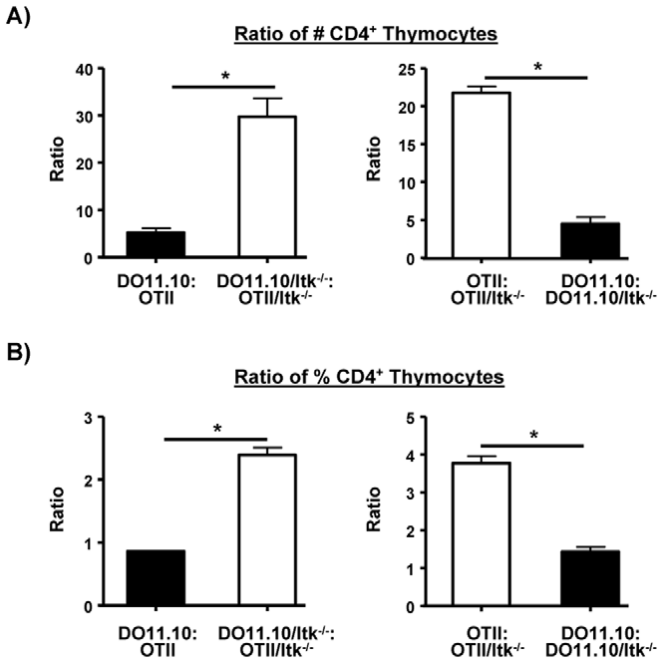
TCR affinity and Itk signals regulate the number of CD4SP cells that develop. (**A**) The ratio of the number of CD4SP thymocytes between the indicated mouse strains were plotted. (B) The ratio of the percentages of CD4SP thymocytes between the indicated mouse strains were plotted.*p<0.05, n = 3–5 mice.

Our results so far analyzing the cross between TCR affinity and Itk expression suggest that higher affinity TCR could partially compensate for the absence of Itk for the development of CD4^+^ T cells. To explore this point further, we analyzed the ratios of the numbers of transgene positive CD4SP thymocytes in the two transgenic mouse strains, and their Itk null counterparts. Comparing the higher affinity DO11.10 mice to OT-II mice revealed that DO11.10 mice generated more CD4SP thymocytes as expected for thymocytes that have higher affinity TCRs and thus better able to be selected (expectation of 1 if DO11.10 and OT-II mice have equal ability to generate CD4SP thymocytes, actual ratio, 5.3, **Fig. 5a**). More strikingly, comparing the transgenic counterparts lacking Itk revealed that DO11.10 mice lacking Itk generated many more CD4SP thymocytes than the OT-II mice lacking Itk (ratio 29.7, **Fig. 5a**). This suggests that the affinity of the DO11.10 TCR had a significant effect in overcoming the absence of Itk.

Comparing the effect of Itk within strains, we found that OT-II mice generated ∼22 fold more CD4SP thymocytes than OT-II mice lacking Itk, while DO11.10 mice had a markedly reduced advantage, only generating ∼5 fold more CD4SP thymocytes than DO11.10 mice lacking Itk (**Fig. 5a**). Similar conclusions could be made if the ratio of the percentages of CD4SP thymocytes are compared (**Fig. 5b**). Together, these data suggest that the affinity of the TCR interacts with those TCR signals regulated by Itk to generate CD4SP thymocytes.

### Reduced TCR Signaling in the Absence of Itk during T Cell Development

**Figure 6:**
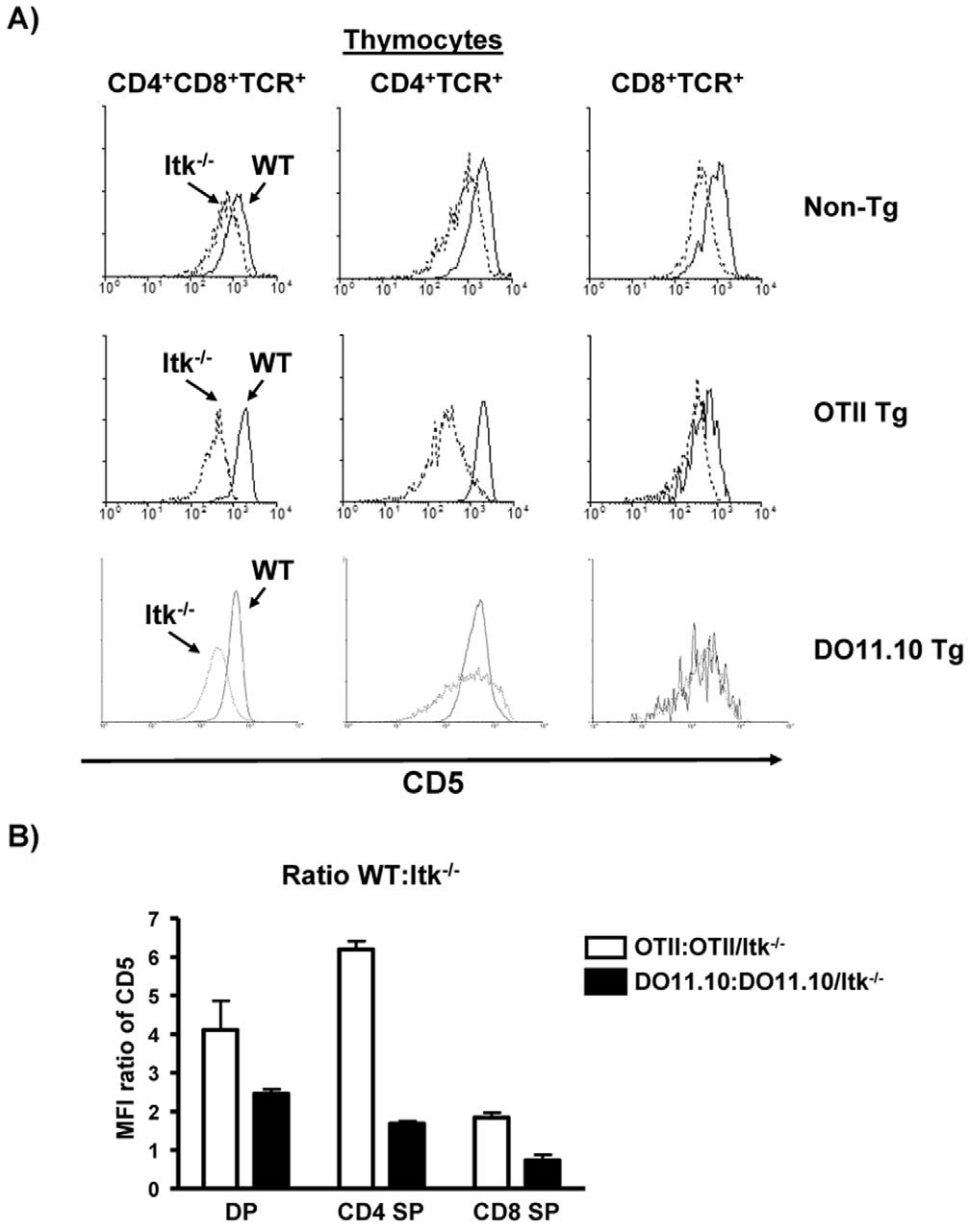
The strength of the TCR signal is reduced in the absence of Itk. (**A**) Thymocytes from non-transgenic WT, Itk^−/−^ mice, TCR transgenic OT-II and OT-II/Itk^−/−^, or TCR transgenic DO11.10 and DO11.10/Itk^−/−^ mice were stained with CD4, CD8α, TCR (or transgenic TCR) and CD5. Histograms of CD5 expression on DP, transgenic TCR^hi^DP, transgenic TCR^hi^CD4SP and transgenic TCR^hi^CD8SP from WT (solid lines), Itk^−/−^ (dashed lines) are shown. A minimum of 6 mice of each genotype with 6–12 weeks of age were analyzed, and representative flow profiles are shown. (**B**) The ratios of the mean fluorescence intensities (MFI) for CD5 for WT:Itk−/− TCR transgenic mice are indicated for double positive and CD4 and CD8 single positive thymocytes. *p<0.05, n = 3–5 mice.

Our results so far suggest that TCR affinity and signals regulated by Itk interact to regulate the development of CD4^+^ T cells. Previous analysis of Itk^−/−^ mice suggests that their T cells receive weak TCR signals, and that Itk may act as an amplifier of T cell receptor signals [9], [10], [11], [12]. CD5 surface expression on mature SP thymocytes and T cells has been found to directly parallel the signaling intensity received by developing thymocytes [34]. Indeed, as shown in figure 6, CD5 expression was lower in non-transgenic total DP thymocytes from Itk^−/−^ mice compared to WT mice (**Fig. 6a**). In addition, in the OT-II transgenic mouse system, CD5 levels were also reduced in the TCR^hi^ DP thymocytes and CD4 SP thymocytes, but less so on CD8 SP thymocytes (**Fig. 6a**). Similar analysis of the DO11.10 transgenic mouse system revealed that CD5 levels were also reduced in the TCR^hi^ DP thymocytes and CD4 SP thymocytes, but not on CD8 SP thymocytes. However, the reduction in CD5 expression was not as profound as that seen in the OT-II system (**Fig. 6a**). Rescaling these data revealed that the ratio of CD5 expression on DP thymocytes between WT:Itk^−/−^ in the two TCR transgenic systems was much higher in the lower affinity OT-II system compared to the DO11.10 system, supporting the view that the differences in development in CD4 SP cells between DO11.10 and OT-II may be due to the level of signals received by developing T cells (**Fig. 6b**). This difference in signal strength also correlated with the level of development of CD8 SP cells between the WT and Itk^−/−^ transgenic systems (i.e. higher ratio of WT:Itk^−/−^ and more development of transgene positive CD8^+^ T cells in the absence of Itk, **Fig. 6b**). These data confirmed that developing DP thymocytes receive weak signals from the TCR in the absence of Itk. This reduced signal may lead to the reduction in CD4^+^ T cell development, and perhaps an increase in CD8^+^ T cells.

### Normal Survival of CD4SP and CD8SP TCR Transgenic Thymocytes in the Absence of Itk

OT-II and OT-II/Itk^−/−^ mice showed the largest difference in CD4SP numbers and percentages both thymus and periphery. We therefore determined if the absence of Itk alters the survival of thymocytes in this background. Since Bcl-2 is a prominent survival factor that has been implicated in preventing programmed cell death in T cells, and is required for prolonged lymphocyte survival following maturation [35], we examined the expression of Bcl-2 in thymocytes from OT-II and OT-II/Itk^−/−^ mice. We found that the levels of Bcl-2 was low in total OT-II DP thymocytes, and was up-regulated in TCR^hi^ DP thymocytes, remaining high in TCR^hi^ CD4 and CD8 SP thymocytes, and OT-II/Itk^−/−^ thymocytes behaved similarly (**Figure S2**). Similarly, there was no difference in annexin V staining between OT-II and OT-II/Itk^−/−^ mice, suggesting no difference in death in thymocytes from these mice (**Figure S3**).

### Itk Regulates the Development of Naïve TCR Transgenic CD4^+^ T Cells

**Figure 7:**
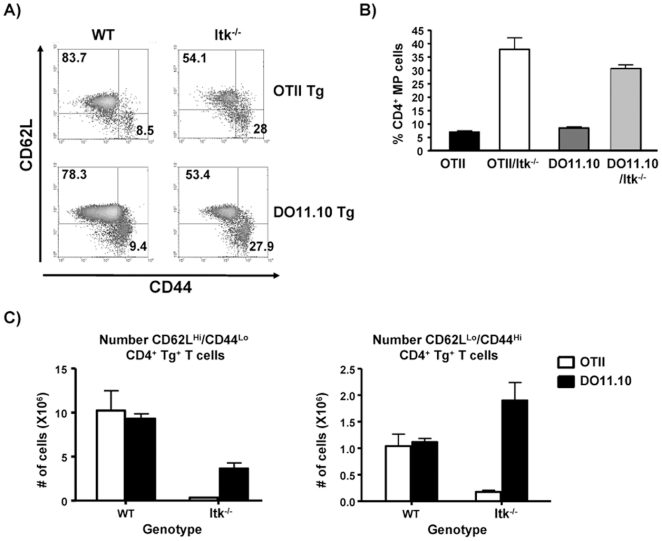
Reduced development of naïve TCR transgene positive CD4^+^ T cells in OT-II/Itk^−/−^ and DO11.10/Itk^−/−^ mice. (**A**) CD4^+^/transgene TCR^+^ splenocytes from OT-II, OT-II/Itk^−/−^, DO11.10 and DO11.10/Itk^−/−^ mice were analyzed for CD44 and CD62L. (**B**) The percentages of TCR transgene, CD4, CD44^hi^ and CD62L^lo^ T cells for each genotype is indicated. p<0.05, n = 3–5. (**C**) The number of TCR transgene, CD4^+^CD62L^lo^CD44^hi^ as well as CD4^+^CD62L^hi^CD44^lo^ T cells for each genotype is indicated.

We and others have recently shown that the absence of Itk preferentially affects the development of naïve CD4^+^ T cells (i.e. CD4^+^CD62L^hi^CD44^lo^), with little effect on populations of CD4^+^ T cells that have an innate memory phenotype (CD4^+^CD62L^lo^CD44^hi^, preformed IFNγ message and ability to rapidly secrete this cytokine [21], [22], [23], [24], [25]). It is not clear if the transgene positive CD4^+^ cells that develop in the absence of Itk are of the “naïve” or “innate memory phenotype”. We therefore analyzed the populations of T cells that developed in the TCR transgenic mice to determine their phenotype. We found that regardless of the affinity of the TCR, the percentage of naïve CD4^+^ T cells (i.e. CD4^+^CD62L^hi^CD44^lo^) in the periphery of these mice was similar if the mice lacked Itk (**Fig. 7a, b**). However, as seen in figures 3 and 4, OTII/Itk^−/−^ mice have much fewer CD4^+^ T cells overall (**Figs. 3d** **,** **4d**).

By contrast both OT-II and DO11.10 mice generate similar numbers of transgene positive CD4^+^CD62L^hi^CD44^lo^ (naïve) T cells, and in both cases, the absence of Itk significantly affects the development of these cells (**Fig. 7c**). However, the lower affinity OT-II transgenic T cells are significantly more affected than the DO11.10 transgenic T cells (**Fig. 7c**). A similar effect is observed when we determined the number of CD4^+^CD62L^lo^CD44^hi^ (memory phenotype) T cells (**Fig. 7c**). Thus the affinity of the TCR has more of an effect on the number of CD4^+^ T cells that develop, than on the percentage of those cells that have a “memory phenotype”. By contrast, Itk affects both parameters.

### Itk Interacts with TCR Affinity to Regulate the Development of “Nonconventional” (or Innate Memory Phenotype) TCR Transgenic CD8^+^ T Cells

**Figure 8:**
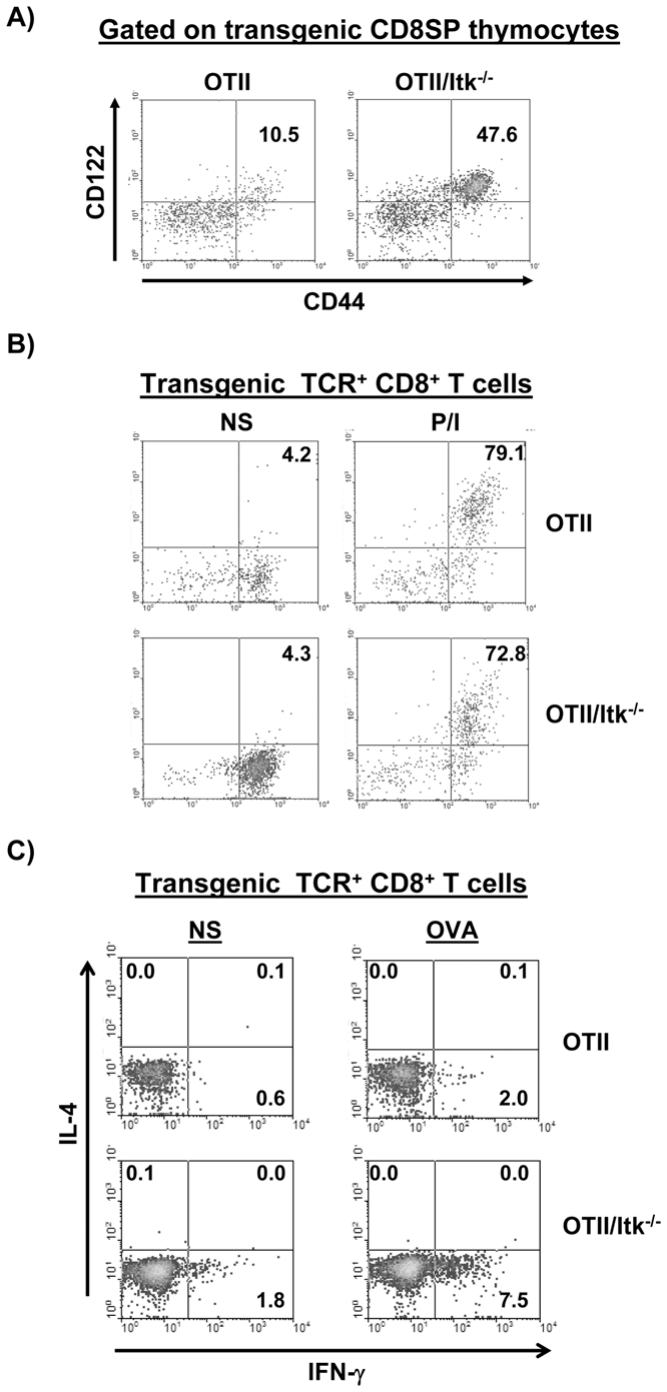
TCR transgene positive CD8 T cells that develop in OT-II/Itk^−/−^ have characteristics of “non-conventional” or “innate memory phenotype” CD8 T cells. (**A**) TCR transgene positive CD8SP thymocytes from OT-II or OT-II/Itk^−/−^ were analyzed for CD44 or CD122 by flow cytometry. (**B**) Splenocytes from OT-II or OT-II/Itk^−/−^ mice were stimulated with PMA and Ionomycin for 6 hours, followed by analysis of TCR transgene positive CD8^+^ T cells for expression of CD44 and IFN-γ. (**C**) Splenocytes from OT-II or OT-II/Itk^−/−^ mice were stimulated with OVA peptide in vitro, followed by analysis of TCR transgene positive CD8^+^ T cells for expression of IL-4 or IFN-γ.

We and others have recently shown that CD8^+^ T cells that have a memory phenotype and show innate function (“non-conventional” T cells) also develop in an Itk independent manner [22], [23], [25]. Here, we also observed that the absence of Itk led to an increase in percentage (and number) of CD8^+^ T cells that bear the OT-II transgenic TCR (**Fig. 3**). However, there was significantly lower percentage of TCR transgene positive CD8^+^ T cells in DO11.10 mice (**Fig. 4**). We therefore further analyzed those cells developing in the OT-II background, and find that the transgenic TCR^hi^CD8^+^ SP thymocytes that develop in OT-II/Itk^−/−^ have phenotypes similar to CD8^+^ SP thymocytes of non-transgenic Itk^−/−^ mice, including high levels of CD44 and CD122, while WT OT-II mice have a much lower frequency of these cells (**Fig. 8a**). These CD8^+^ T cells were also able to rapidly produce IFN-γ upon ex vivo stimulation with PMA/Ionomycin (**Fig. 8b**).

These TCR^hi^ CD8^+^ T cells could respond to the MHC class II restricted antigen (OVA_323–339_ peptide) recognized by the transgenic TCR when cultured in the presence of this peptide (total splenocytes were used due to the difficulty in isolating the small number of TCR^hi^CD8^+^ T cells from these TCR transgenic mice). After 7 days of culture cytokine secretion from the transgenic TCR^hi^CD8^+^ cells was analyzed by restimulation of these cells with the OVA peptide. The transgenic TCR^hi^CD8^+^ cells from OT-II/Itk^−/−^ mice, but not those cells from OT-II mice, produced IFN-γ after MHC class II-restricted OVA stimulation (**Fig. 8c**). It is possible that transgenic Itk^−/−^ TCR^hi^CD8^+^ cells did not respond to the specific peptide stimulation, but instead to cytokines and other factors produced by activated CD4^+^ T cells in the culture. We think this is unlikely as WT CD4^+^ T cells respond very well to the OVA peptide, yet the CD8^+^ Tg^+^ T cells did not respond to peptide restimulation in those cultures. These CD8^+^ T cells are restimulated with the OVA peptide followed by analysis of intracellular IFN-γ, indicating that the Itk^−/−^ cells can respond to this peptide for cytokine secretion, whereas WT CD8^+^ T cells cannot. We also note that the few CD8^+^ transgene positive cells found in WT OT-II that produce IFN-γ in response to PMA/Ionomycin stimulation do not respond to the OVA peptide when restimulated in vitro (**Fig. 8b, c**). These data suggest that the transgenic TCR^hi^CD8^+^ T cells that develop in OT-II/Itk^−/−^ mice are likely MHC class-II restricted and can be activated by class II-restricted peptide, but bear the hallmarks of “non-conventional” or “innate memory phenotype” CD8 T cells.

### Reduced Expression of the CD4 Lineage Commitment Factor, Th-POK, in Itk Null DP Thymocytes

**Figure 9:**
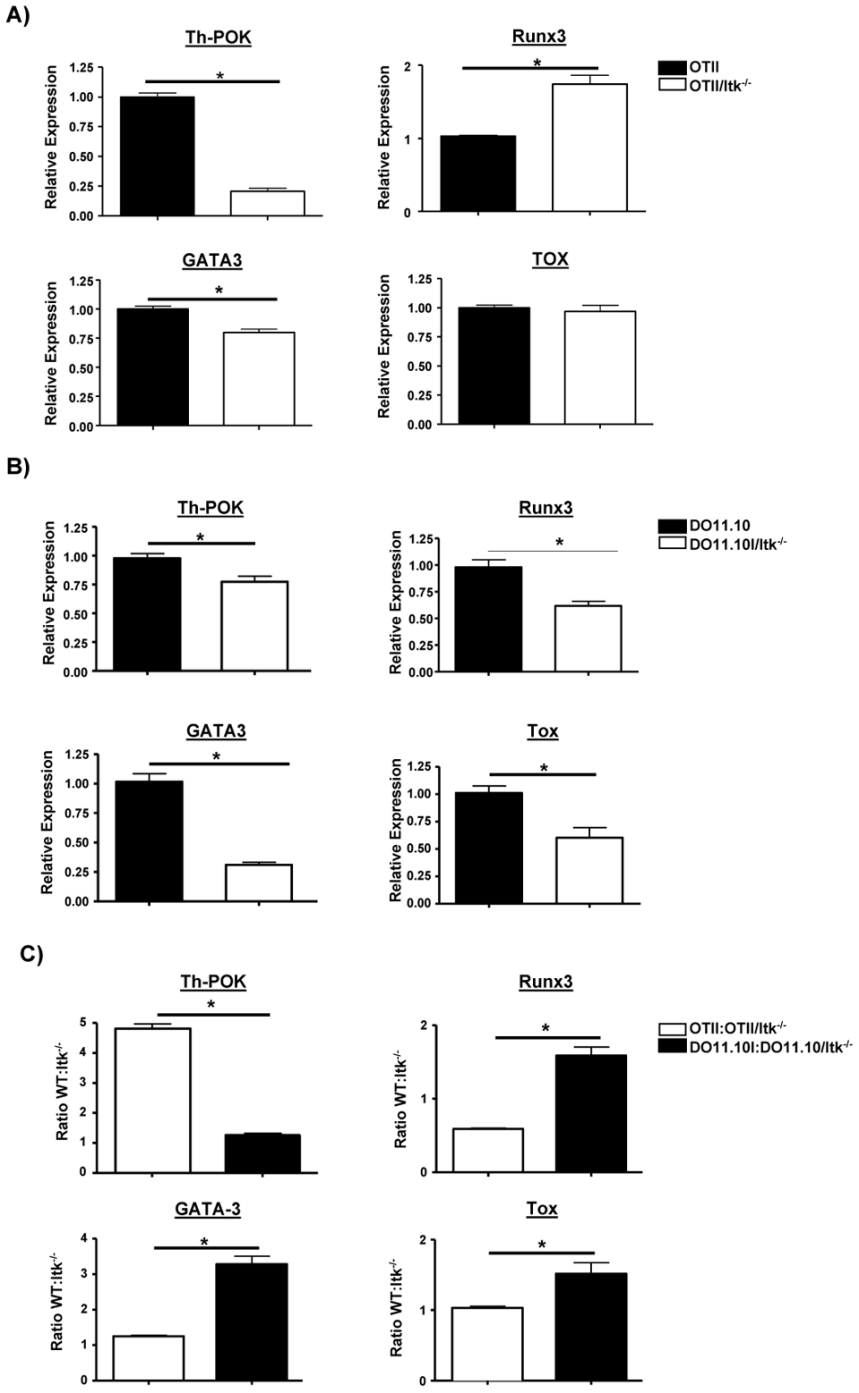
Itk mediated signals regulate the expression of the CD4 lineage commitment factor Th-POK. (**A**) Quantitative real-time RT-PCR for Th-POK, TOX, Runx3 and GATA3 in sorted transgenic TCR^hi^ DP thymocytes from OT-II and OT-II/Itk^−/−^ mice. Data are corrected for GAPDH expression and expressed as fold over mRNA of the transgenic TCR^hi^ DP derived from OT-II mice, which was set at 1 (n = 3, *p<0.05). The experiment was repeated 2X with the same results. (**B**) Quantitative real-time RT-PCR for Th-POK, TOX, Runx3 and GATA3 in sorted transgenic TCR^hi^ DP thymocytes from DO11.10 and DO11.10/Itk^−/−^ mice Data are corrected for GAPDH expression and expressed as fold over mRNA of the transgenic TCR^hi^ DP derived from DO11.10 mice, which was set at 1 (n = 3, *p<0.05). The experiment was repeated 2X with the same results. (**C**) Data used to generate (A) and (B) were rescaled to generate ratios of either OT-II:OTII/Itk^−/−^ or DO11.10:DO11.10/Itk^−/−^ mRNA expression levels for Th-POK, Runx3, GATA3 and TOX.

A large body of evidence supports the view that the amount of TCR signaling determines the lineage commitment decisions for development of the CD4 and CD8 T cells. The nature of the signals that affect changes in gene expression that specify the CD4/CD8 T cell lineage commitment and enforcement has become a major goal of work in this area. Several transcription factors essential for CD4/CD8 T cell lineage commitment have been identified, including the Runx factors, as well as TOX, GATA3 and Th-POK [36], [37], [38], [39], [40], [41], [42], [43]. Among these transcription factors, Th-POK has been identified as a master enforcer of CD4 commitment [44], [45], [46], [47]. Th-POK is a Zn finger transcription factor, and is expressed specifically in the CD4 lineage. Enforced constitutive expression of Th-POK not only leads to normal development of MHC class II-restricted T cells to the CD4 lineage but also causes redirection of MHC class I-restricted cells to the CD4 lineage [46], [48]. Notably, however, signals that regulate the expression of Th-POK and thus CD4 lineage commitment are less clear. Our data suggest that the absence of Itk affects the strength of signal through the TCR and thus the development of CD4SP (and potentially CD8SP) in an environment of enforced MHC class II-restriction. To determine if these reduced signals regulate the transcription factors that modulate CD4/CD8 lineage commitment, we analyzed sorted transgenic TCR^hi^ DP thymocytes from OT-II, OT-II/Itk^−/−^, DO11.10 and DO11.10/Itk^−/−^ mice, and determined the expression levels of different transcription factors using quantitative real-time RT-PCR. As shown in figure 9, Th-POK expression in OT-II/Itk^−/−^DP thymocytes was significantly lower than those from OT-II mice (**Fig. 9a**). By contrast, the level of Th-POK expression in DO11.10/Itk^−/−^ mice was closer to that seen in WT DO11.10 mice (**Fig. 9b**), in concordance with the differences in affinity and signal strength (as detected by CD5 expression, see **Fig. 6**). Examining the ratio of expression between WT and Itk^−/−^ revealed that like CD5 expression, expression of Th-POK was regulated by Itk and TCR affinity (**Fig. 9c,**i.e. the ratio is larger between the lower affinity OT-II and OT-II/Itk^−/−^ DP thymocytes). Given the role of this factor in regulating the development of CD4 SP thymocytes, these data suggest that the strength of the TCR signal that thymocytes receive regulate the expression of Th-POK, and thus CD4^+^ T cell development. Analysis of the percentage of CD69^+^ cells in WT and OT-II and OT-II/Itk^−/−^ DP thymocytes did not reveal any differences in expression, suggesting that differences in DP thymocytes that have received differentiation signals (and are CD69^+^), does not explain the observed differences in Th-POK expression (see **figure S4**).

We also analyzed the levels of Runx3, which has been shown to bind to the CD8 enhancer and contribute to CD8 expression in CD8SP and mature CD8 lineage T cells [38]. Runx3 has also been suggested to negatively regulate Th-POK expression [49]. Figure 9 shows that the level of Runx3 is higher in OT-II/Itk^−/−^ DP thymocytes compared to cells from OT-II (**Fig. 9a**). By contrast DO11.10/Itk^−/−^ DP thymocytes had the reverse pattern, with the WT counterparts expressing higher levels of Runx3 compared to the Itk null animals, a point made more evident when the WT:Itk^−/−^ ratios are compared (**Fig. 9c**). The significance of this difference is unclear, since Th-POK expression and genetic programming for CD4 T cell development is inhibited by Runx-dependent silencer activity [49]. Similarly, analysis of GATA3 revealed that the difference in expression between OT-II and OT-II/Itk^−/−^ DP thymocytes was smaller compared to the higher affinity DO11.10 and DO11.10/Itk^−/−^ thymocytes (**Fig. 9a, b & c**). GATA3 has been shown to regulate CD4 T cell development and may regulate Th-POK expression [41], [42], [43]. By contrast, while no significant difference was observed in levels of TOX in the OT-II transgenic system, TOX expression was reduced in the DO11.10 mice compared to DO11.10/Itk^−/−^ mice (**Fig. 9a, b, c**).

**Figure 10:**
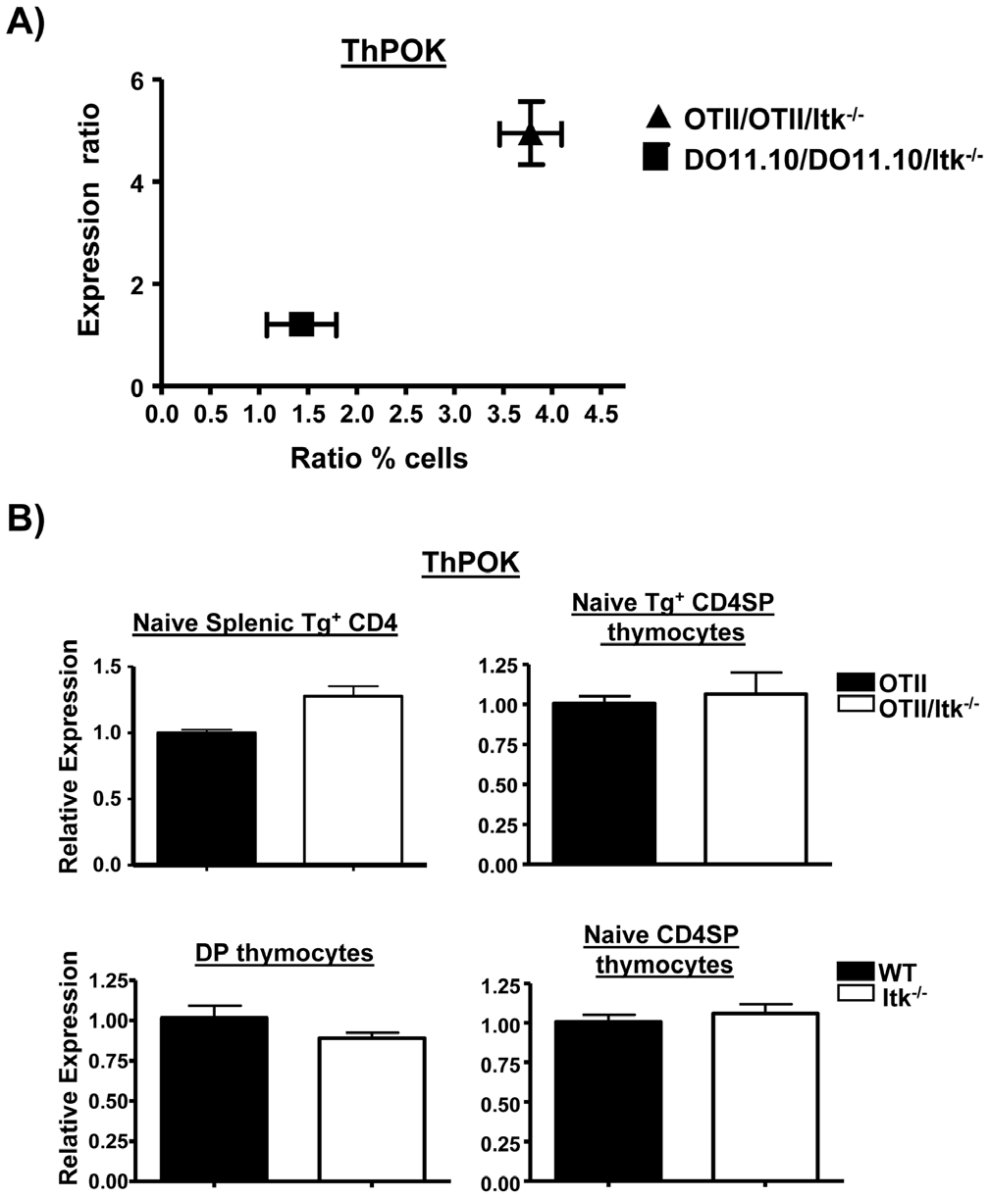
Itk mediated signals intersect with TCR affinity to regulate the expression of Th-POK and the development of CD4^+^ T cells. (**A**) The ratios and percentages of CD4SP thymocytes in OT-II:OTII/Itk^−/−^ (triangles) and DO11.10:DO11.10/Itk^−/−^ (squares) mice were plotted against the corresponding ratios of the expression of Th-POK in their DP thymocyte population. (**B**) Quantitative real-time RT-PCR for Th-POK in sorted transgenic TCR^+^ naïve splenic CD4^+^ T cells (top left), sorted transgenic TCR^+^ naïve CD4SP thymocytes (top right), sorted non-transgenic DP thymocytes (bottom left) or sorted non-transgenic naïve splenic CD4^+^ T cells (bottom right). Data are corrected for GAPDH expression and expressed as fold over mRNA of the corresponding WT mice, which was set at 1 (n = 3 with no significant difference observed).

In order to further explore the intersection between TCR affinity and Itk signals in regulating CD4SP development, we rescaled the data for Th-POK, and compared the ratios of expression Th-POK to the ratios of CD4SP in the two transgenic systems. Revealingly, there was a significant relationship between the ratio of expression of Th-POK in the TCR transgenic thymocytes, and their development of CD4SP cells. Thus the larger difference in expression of Th-POK between OT-II and OT-II/Itk thymocytes was associated a larger difference in the percentage of CD4SP cells compared to the DO11.10 system (**Fig. 10a**). Together, these data suggest that a combination of TCR affinity and signals derived from Itk regulate Th-POK expression, thus modulating the development of CD4^+^ T cells.

We also determined Th-POK expression in conventional (CD44^lo^/CD62L^hi^) CD4SP thymocytes and peripheral CD4^+^ T cells. We find that unlike DP thymocytes, there was no difference in expression of Th-POK (**Fig. 10b**). Our interpretation of this finding is that those Itk^−/−^ cells that express a threshold level of Th-POK are allowed to differentiate, but this frequency is much lower than that seen in the WT mice, hence the reduced numbers of mature CD4SP in the absence of Itk. Indeed, analysis of Th-POK expression in non-transgenic WT and Itk^−/−^ DP thymocytes revealed no significant differences in expression, perhaps due to the range of TCRs with different affinities in a non-transgenic background that may allow signaling of appropriate Th-POK expression (**Fig. 10b**).

### Elevated Expression of Runx Targets Genes in Itk Null CD4SP Thymocytes

**Figure 11:**
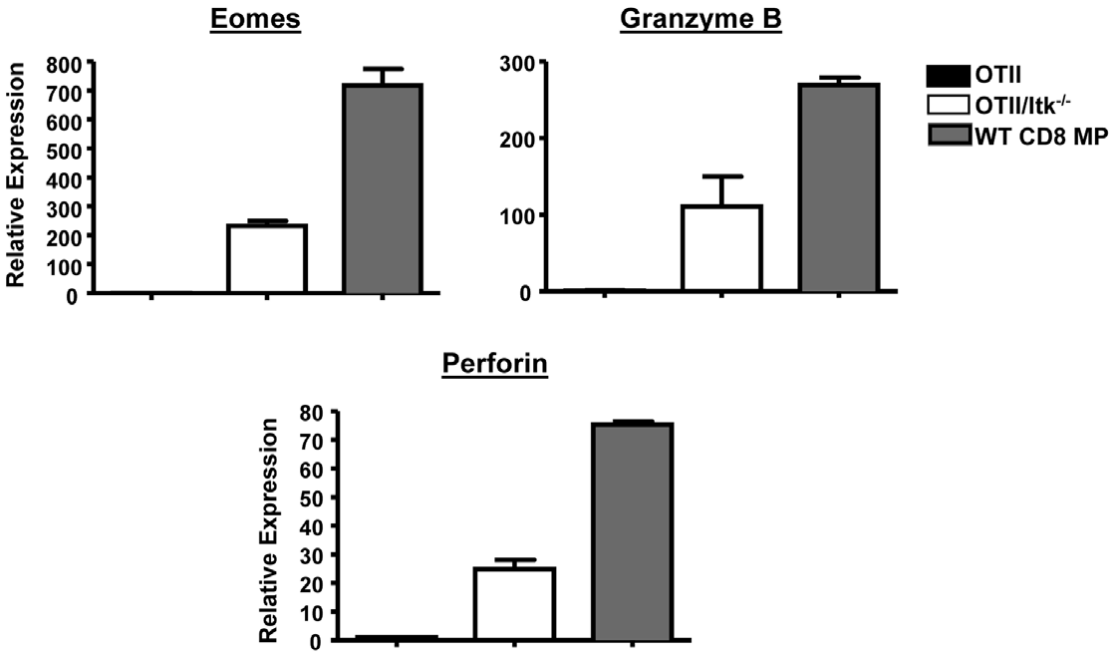
Itk mediated signals intersect with TCR affinity to regulate Runx3 regulated genes in naïve or conventional CD4^+^ T cells. Quantitative real-time RT-PCR for Perforin, Granzyme B and Eomesodermin in sorted transgenic TCR^hi^ CD4SP thymocytes from OT-II and OT-II/Itk^−/−^ mice Data are corrected for GAPDH expression and expressed as fold over mRNA of the respective OT-II cells, which was set at 1 (n = 3, *p<0.05). Expression of these genes in sorted WT memory phenotype CD8^+^ T cells are shown as a reference.

The altered expression of Th-POK in the absence of Itk, and the elevated Runx3 expression in the low affinity OT-II TCR transgenic system prompted us to determine whether downstream targets of Runx3 are affected in the absence of Itk. We therefore analyzed the expression of Eomesodermin, Granzyme B and Perforin in conventional (CD44^lo^CD62L^hi^) CD4SP thymocytes in the presence and absence of Itk in the OT-II transgenic system (to rule out potential contributions from Memory Phenotype (MP) CD4SP cells [27]). We find that the absence of Itk in the CD4SP OT-II transgenic thymocytes results in elevated expression of Eomesodermin, Granzyme B and Perforin, suggesting that reduced Th-POK expression may unleashed the function of Runx proteins in driving the expression of these genes (**Fig. 11**).

## Discussion

This study was designed to examine whether CD4/CD8 lineage choices are affected by the reduced TCR signals in the absence of Itk. We analyzed Itk^−/−^ mice crossed to mice carrying two TCRs reactive against ovalbumin, the low affinity MHC class II-restricted transgenic TCR (OT-II) and the higher affinity (50 fold) MHC class II-restricted transgenic TCR (DO11.10). Our results show that dependent on the affinity of the TCR, Itk affects the development of naïve CD4^+^ T cells. Our results also suggest that this occurs via Itk mediated (direct or indirect) regulation of Th-POK expression, suggesting that the strength of the TCR signal modulates Th-POK expression and thus CD4^+^ T cell development. Finally, our results suggest that by modulating Th-POK expression, Itk derived signals affect the expression of CD8 patterning genes such as Runx3, which regulates the expression of Eomesodermin, Granzyme B and Perforin in naïve or conventional CD4SP thymocytes, altering the potential response of these T cells. Finally, our results suggest that at significantly reduced TCR affinity, “non conventional” CD8^+^ T cells begin to develop.

Berg and colleagues have previously used a TCR transgenic approach to analyze the role of Itk in CD4 and CD8 T cell commitment [18]. They analyzed three different lines of MHC class II-restricted TCR transgenics, 2B4, 5C.C7 and AND, all lacking Itk and found a significant difference in the number of CD4SP T cells, although there was not much difference in CD8SP T cells based on the affinity of the TCRs analyzed (2B4, 5C.C7 and AND). Based on our results, we suggest that the strength of the TCR intersects with Itk signals to reach specific thresholds (such as Th-POK expression) for CD4 lineage commitment/enforcement. Those few Itk null CD4^+^ T cells that receive a high enough signal could express the appropriate levels of Th-POK and are allowed to continue their differentiation, which occurs at much reduced frequency in the absence of Itk. Previous analysis of the DO11.10 MHC class II-restricted TCR in the absence of active Lck did not reveal altered CD4 and CD8 development [50]. However, Alberola-Ila and colleagues showed that functional CD8^+^ T cells develop in mice transgenic for the MHC class II-restricted AND TCR when Lck activity was reduced, suggesting that the magnitude of the reduction in TCR signals may be important for this effect [4], [51].

Our analysis of genes that regulate the expression of CD4 and CD8 lineage commitment in double positive thymocytes revealed that Itk mediated signals regulate the expression of Th-POK, previously identified as a major enforcer of CD4 T cell commitment [24]–[26] [43]. Similarly, the expression of GATA3, another regulator of CD4 T cell commitment was significantly reduced in low affinity OT-II/Itk^−/−^ TCR transgenic DP thymocytes [34], [35]. However, the expression of TOX, which may also regulate Th-POK expression was not altered. By contrast, the higher affinity DO11.10 system had less of a reduction in Th-POK and a larger reduction in expression of Runx3, GATA3 and TOX. The significance of these differences is not clear. Nevertheless, these data suggest that perhaps there is an absolute amount of signal that is required to induce or maintain the expression of Th-POK, following which CD4 lineage commitment is fixed. However, in the presence of a low affinity TCR and reduced signals in the absence of Itk, the frequency of cells that receive such signals leading to Th-POK expression is reduced. Our finding that WT and Itk^−/−^ conventional (CD44^lo^/CD62L^hi^) transgenic CD4SP thymocytes and peripheral CD4^+^ T cells express similar levels of Th-POK supports this view, and suggests that those Itk^−/−^ cells that express threshold levels of Th-POK for CD4 differentiation are allowed to proceed, but that this frequency is much lower than that seen in the WT mice, hence the reduced numbers of mature CD4SP in the absence of Itk. The lower levels of Th-POK expressed in those remaining Itk^−/−^ developing DP thymocytes may now allow enhanced expression of Runx3, and accompanying increases in Runx3 regulated genes Eomesodermin, Granzyme B and Perforin [52], [53], [54]. Of course, it is possible that Itk derived signals combine with TCR affinity to negatively regulate Runx3 such that in their absence, Runx3 expression is elevated, suppressing Th-POK expression and reducing CD4^+^ T cell development [55]. Either model would predict that on a non-transgenic background, the affinity of the TCRs expressed by Itk null CD4^+^ T cells would tend to be of higher affinity so as to overcome the reduction in TCR signals in the absence of Itk.

We and others have previously shown that Itk is required for the development of conventional CD8^+^ T cells, while leaving intact “non-conventional” CD8^+^ T cells that have a memory phenotype and exhibit innate function [22], [23], [25]. In this paper, we show that memory phenotype CD8^+^ T cells can still develop even in the presence of a fixed MHC class II restricted TCR transgene that drives CD4^+^ T cell development, suggesting that the development of these CD8^+^ MP cells may be a consequence of impaired TCR signaling (although it is possible that these TCR transgene positive CD8^+^ T cells also carry endogenous TCRs and were selected on those receptors). Note that MHC class I restricted OT-1/Itk^−/−^ mice develop normal conventional CD8^+^ T cell due to efficient selection in the thymus of T cells carrying this transgenic TCR [23]. Alberola-Ila and colleagues' previous work on the influence of Lck and Ras/MAP kinase pathways on CD4 and CD8 T cell commitment suggested that MHC class II-restricted CD8^+^ T cells that develop in an environment of reduced Lck signals behave as competent cytotoxic T cells, able to kill target cells [4]. It was not determined whether the CD8^+^ T cells that developed in those systems were all conventional CD8 T cells. The CD8^+^ T cells that develop in OT-II/Itk^−/−^ mice also express high levels of perforin and granzyme B, and can degranulate following stimulation (data not shown), all characteristics of cytotoxic CD8^+^ T cells. It is possible that the CD8^+^ T cells that develop under conditions of reduced Lck activity are also innate memory phenotype cells. In the absence of Itk and in the environment of a low affinity TCR such as the OT-II TCR, these cells may develop due to a default differentiation program to CD8SP cells that results from reduction in Th-POK expression and increase in Runx3 expression, as has been suggested by the observed increase in CD8SP development when Th-POK levels are reduced [56]. Whether these cells would develop in the context of the transgene and knockout crossed onto a RAG null background is not clear.

Although the discovery of Th-POK as a major commitment factor for CD4 lineage commitment represented a huge advance in our understanding of CD4 T cell development, signals that regulate the expression of the factor are unknown. Our work reported here shows that Itk plays a role in regulating this factor, thus regulating the development of CD4^+^ T cells. In addition, our work connects signals emanating from the TCR, via Itk, and the expression of specific genes that regulate lineage commitment, suggesting a possible mechanism by which TCR signals can regulate CD4/CD8 commitment.

High levels of Lck and ERK MAPK has been shown to lead to enhanced CD4 T cell development to the detriment of CD8 T cell development [4], [5], while reduced activity of Lck, ZAP70 and ERK leads to reduced CD4 T cell development, leaving CD8 T cell development intact [4], [5], [6]. Our data here suggest that a Lck-Itk-MAP kinase signaling pathway in double positive thymocytes may modulate the development of CD4^+^ T cells, with perhaps default development of CD8 T cells (with of course consequences for the development of conventional vs. non-conventional or innate memory phenotype CD8 T cell development). Our data also suggest that components of the TCR signaling pathway may affect the lineage choices of T cells dependent on the affinity of the TCR. Final decisions on lineage choice may thus be determined by the combination of TCR affinity and strength of the TCR signals with absolute levels of signaling determining lineage commitment choices.

## Materials and Methods

### Mice

We used WT or Itk^−/−^ mice all on a C57BL/6 background, between 6–12 weeks of age, which were kept in specific pathogen free conditions. OT-II and DO11.10 transgenic mice were from Jackson Labs (Bar Harbor, Maine) and bred in our lab. OT-II/Itk^−/−^ mice were generated by crossing Itk^−/−^ mice to OT-II transgenic mice. These mice were intercrossed, then backcrossed >5 generations. DO11.10/Itk^−/−^ mice were generated by crossing Itk^−/−^ mice on a BALB/c background (kind gift of Dr. Deborah Fowell, University of Rochester [57]), to DO11.10 transgenic mice. *Tg(Lck-ItkΔKin)Itk^−/−^* and *Tg(CD2-Itk)Itk^−/−^* mice were generated previously in our lab and were backcrossed to the C57BL/6 background >10 generations [27], [28]. All experiments were approved by the IACUC at Pennsylvania State University.

### Antibodies and Flow Cytometry

Cells were incubated for half an hour at 4°C with antibodies in 100 µl PBS/2% FBS, followed by two washes in PBS/2% FBS for surface staining. The following antibodies were purchased from BD Pharmingen and used as suggested by the manufacturer (BD Pharmingen, San Diego, California): anti-CD8-FITC, Vα2-PE, Vβ5-FITC, CD44-Cychrome, CD5-PE, CD45.2-FITC, IL-4-PE, CD122-PE, and IFNγ-PE-Cy7. Anti-CD4-ECD was from Invitrogen (Carlsbad, California) and anti-murine-Bcl-2-PE was from Santa Cruz Biotechnology (Santa Cruz, California). Intracellular staining of Bcl-2 was carried out using a Fixation/Permeabilization kit from BD Pharmingen (San Diego, California). Cells were analyzed using a FC500 Cytometer from Beckman Coulter (Fullerton, California).

### Quantitative Real-Time PCR Analysis

TCR^hi^ DP thymocytes, CD4SP thymocytes or naïve splenic CD4^+^ were sorted from the thymus of transgenic OT-II, OT-II/Itk^−/−^, DO11.10 and DO11.10/Itk^−/−^ mice using a Cytopeia Cell Sorter (Cytopeia Inc., Seattle, WA). Total RNA was prepared from sorted cells using RNease mini kit (Qiagen Sciences, Maryland). cDNA was generated using You Prime First-Strand beads (GE Healthcare, Buckinghamshire, UK), and quantitative PCR was performed using primer/probe sets for Th-POK, TOX, Runx3, GATA3, Eomesodermin, Granzyme B and Perforin (Applied BioSystems, Branchburg, New Jersey), with GAPDH as a housekeeping gene. Data was analyzed using the ΔΔ Comparative CT (threshold cycle) method and normalized to GAPDH and relative to a calibrator sample. The relative gene expression levels were then determined by comparing to the expression found in the transgenic TCR^hi^ OT-II or TCR^hi^ DO11.10 DP populations, which were set as 1.

### In Vitro Analysis of Cytokine Secretion

Spleens were removed from mice and total splenocytes from OT-II and OT-II/Itk^−/−^ mice were cultured with 1 µg/ml OVA_323–339_ peptide (Research Genetics, Huntsville, AL) for 7 days. Dead cells were removed using lympholyte-M (Cedarlane Laboratories, Burlington, NC), and viable cells then stimulated with OVA (1 µg/ml) for 6 hours in the presence of Brefeldin A (10 µg/ml), followed by analysis of cytokine secretion by intracellular cytokine staining. In some cases, cells were stimulated with PMA and Ionomycin for 6 hours in the presence of Brefeldin A (10 µg/ml), followed by analysis of cytokine secretion by intracellular cytokine staining.

### Statistical Analysis

Data was analyzed using Prizm, and significance determined using Students' *t* test, with a value of p<0.05 considered statistically significant.

## Supporting Information

Figure S1 Expression of transgenic TCR on OT-II and D011.10 thymocytes. Thymocytes from TCR transgenic OT-II, OT-II/Itk^−/−^, D011.10 or D011.10/Itk^−/−^ mice were stained with antibodies against CD4, CD8α and TCRVα2 (for OT-II) or anti-KJ-126 (for D011.10), and TCR transgene expression analyzed on CD4SP and CD8SP cells. WT backgrounds are indicated by solid lines and Itk^−/−^ background by dashed lines.
(3.32 MB TIF)

Figure S2 Normal expression of Bcl-2 in TCR transgenic CD4 and CD8 SP thymocytes in OT-II mice the absence of Itk. Thymocytes from TCR transgenic OT-II and OT-II/Itk^−/−^ were stained with CD4, CD8α, TCRVβ5 and intracellular Bcl-2. Histograms of Bcl-2 expression on DP, transgenic TCR^hi^DP, transgenic TCR^hi^CD4SP and transgenic TCR^hi^CD8SP from OT-II (solid line) and OT-II/Itk^−/−^ (dashed line) are shown (filled histogram: nonspecific isotope staining). A minimum of 10 mice of each genotype with 6–12 weeks of age were analyzed, and representative flow profiles are shown.
(1.46 MB TIF)

Figure S3 Normal survival of TCR transgenic CD4 and CD8 SP thymocytes in OT-II mice in the absence of Itk. Thymocytes from transgenic OT-II and OT-II/Itk^−/−^ were stained with CD4, CD8α and TCRVβ5, along with 7AAD and Annexin V. Histograms of Annexin V expression on 7AAD^-^ DP, 7AAD^-^ transgenic TCR^hi^ DP, CD4SP and CD8SP are shown. The percentage of Annexin V^+^ cells for each subset is presented. A minimum of 4 mice of each genotype with 6–12 weeks of age were analyzed, and representative flow diagrams are shown.
(2.15 MB TIF)

Figure S4 CD69 expression and gene expression profiles of naïve (conventional) CD4SP thymocytes in the OT-II transgenic system. Thymocytes from TCR transgenic OT-II and OT-II/Itk^−/−^ were stained with antibodies against CD4, CD8α and CD69. Representative histograms of CD69 expression on gated CD4/CD8 DP thymocytes are shown for OT-II (solid line) and OT-II/Itk^−/−^ (dashed line).
(3.91 MB TIF)
