# Supplementary figures and images for: Itk Derived Signals Regulate the Expression of Th-POK and Controls the Development of CD4+ T Cells
Source: PLoS One. 2010 Jan 26;5(1):e8891. doi: 10.1371/journal.pone.0008891 (PMC2811181; doi:10.1371/journal.pone.0008891)

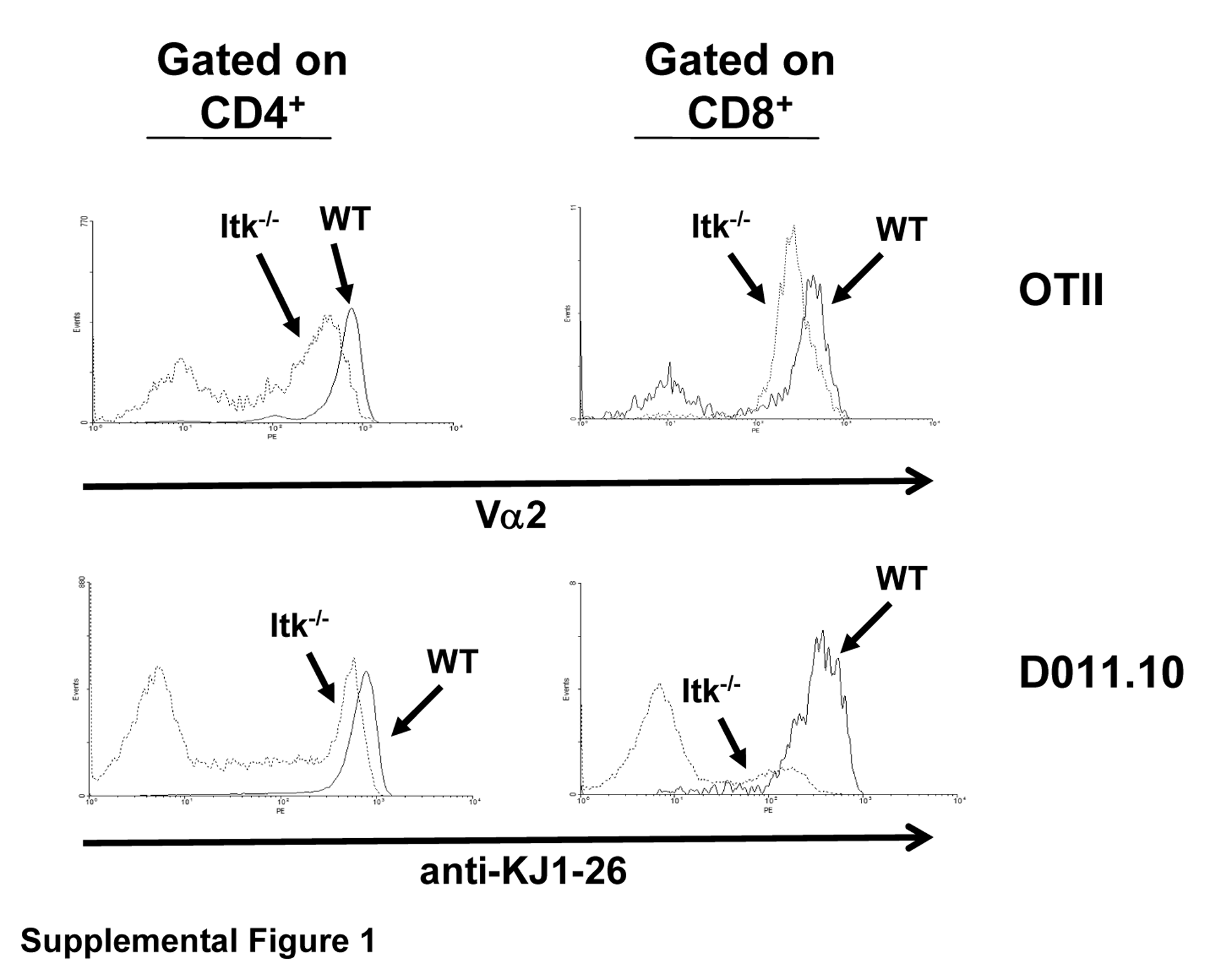

Supplement: Figure S1 — Expression of transgenic TCR on OT-II and D011.10 thymocytes. Thymocytes from TCR transgenic OT-II, OT-II/Itk−/−, D011.10 or D011.10/Itk−/− mice were stained with antibodies against CD4, CD8α and TCRVα2 (for OT-II) or anti-KJ-126 (for D011.10), and TCR transgene expression analyzed on CD4SP and CD8SP cells. WT backgrounds are indicated by solid lines and Itk−/− background by dashed lines. (3.32 MB TIF) [file pone.0008891.s001.tif]

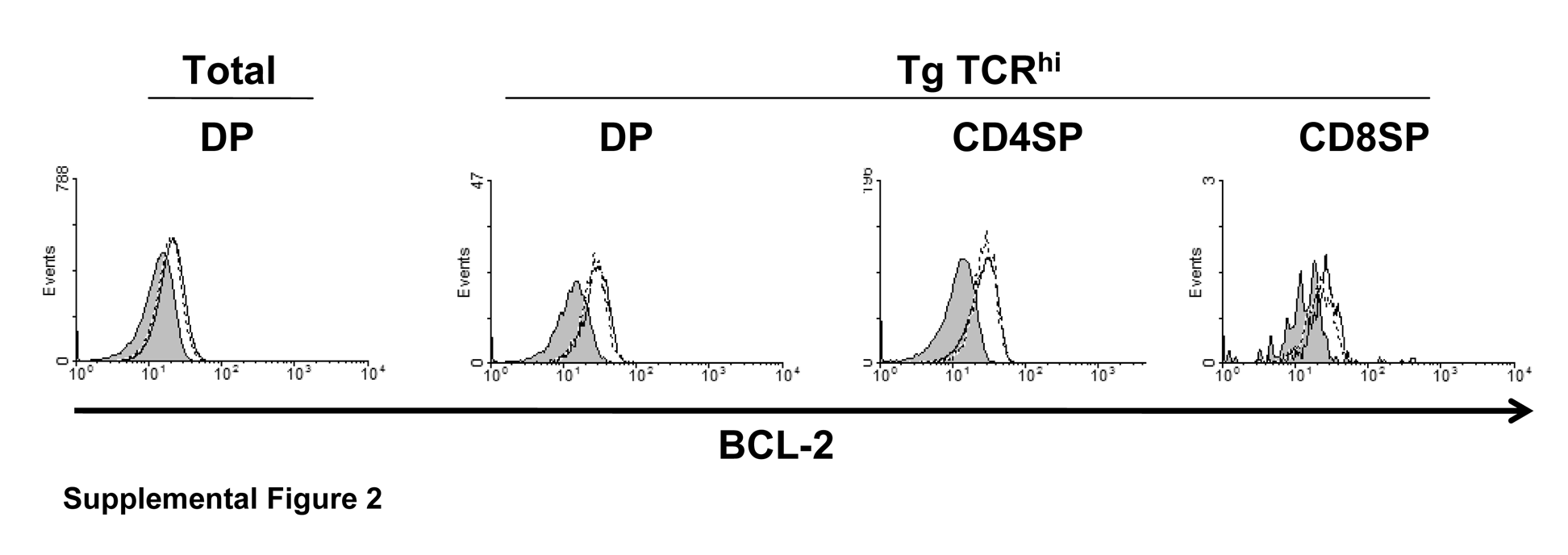

Supplement: Figure S2 — Normal expression of Bcl-2 in TCR transgenic CD4 and CD8 SP thymocytes in OT-II mice the absence of Itk. Thymocytes from TCR transgenic OT-II and OT-II/Itk−/− were stained with CD4, CD8α, TCRVβ5 and intracellular Bcl-2. Histograms of Bcl-2 expression on DP, transgenic TCRhiDP, transgenic TCRhiCD4SP and transgenic TCRhiCD8SP from OT-II (solid line) and OT-II/Itk−/− (dashed line) are shown (filled histogram: nonspecific isotope staining). A minimum of 10 mice of each genotype with 6–12 weeks of age were analyzed, and representative flow profiles are shown. (1.46 MB TIF) [file pone.0008891.s002.tif]

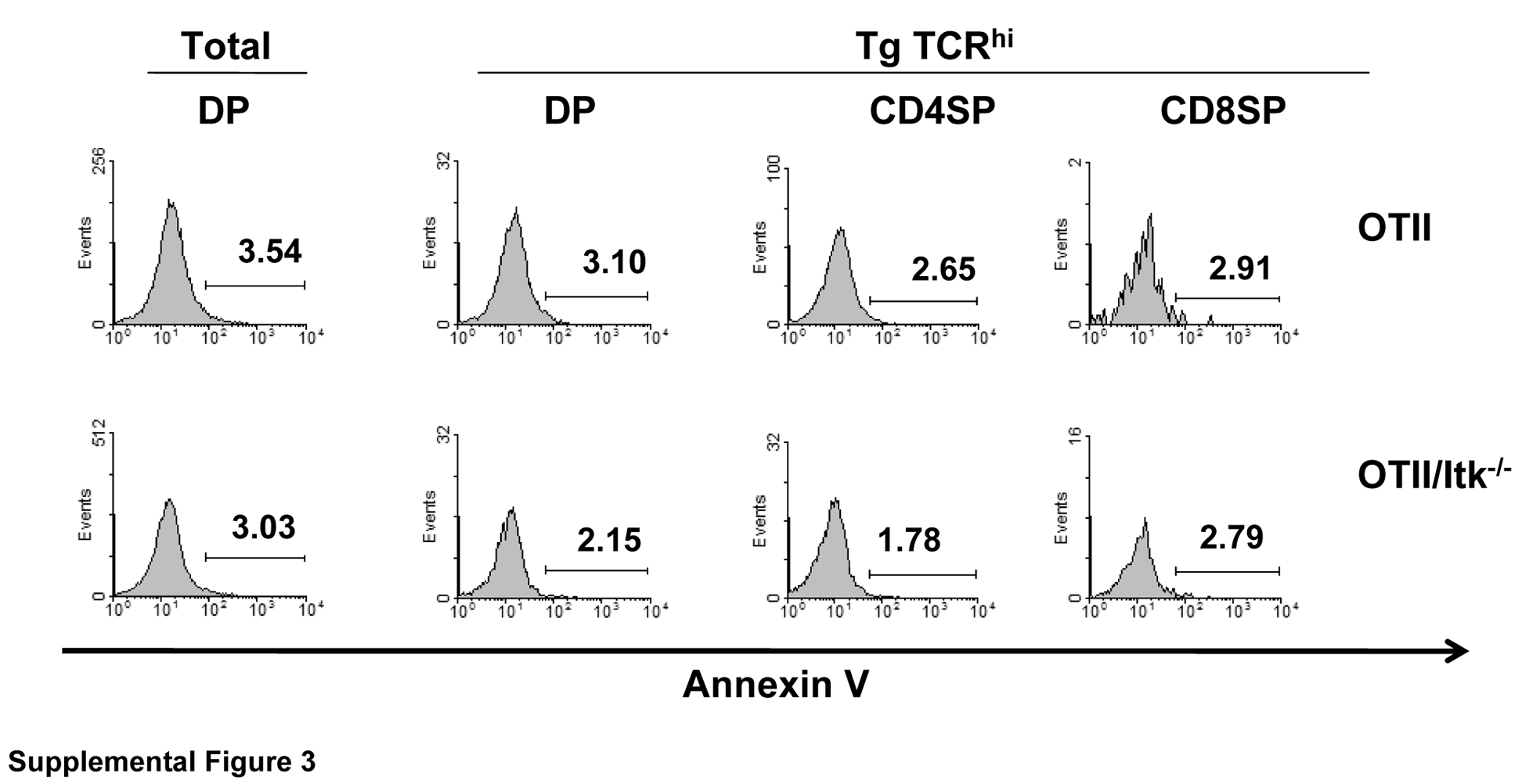

Supplement: Figure S3 — Normal survival of TCR transgenic CD4 and CD8 SP thymocytes in OT-II mice in the absence of Itk. Thymocytes from transgenic OT-II and OT-II/Itk−/− were stained with CD4, CD8α and TCRVβ5, along with 7AAD and Annexin V. Histograms of Annexin V expression on 7AAD- DP, 7AAD- transgenic TCRhi DP, CD4SP and CD8SP are shown. The percentage of Annexin V+ cells for each subset is presented. A minimum of 4 mice of each genotype with 6–12 weeks of age were analyzed, and representative flow diagrams are shown. (2.15 MB TIF) [file pone.0008891.s003.tif]

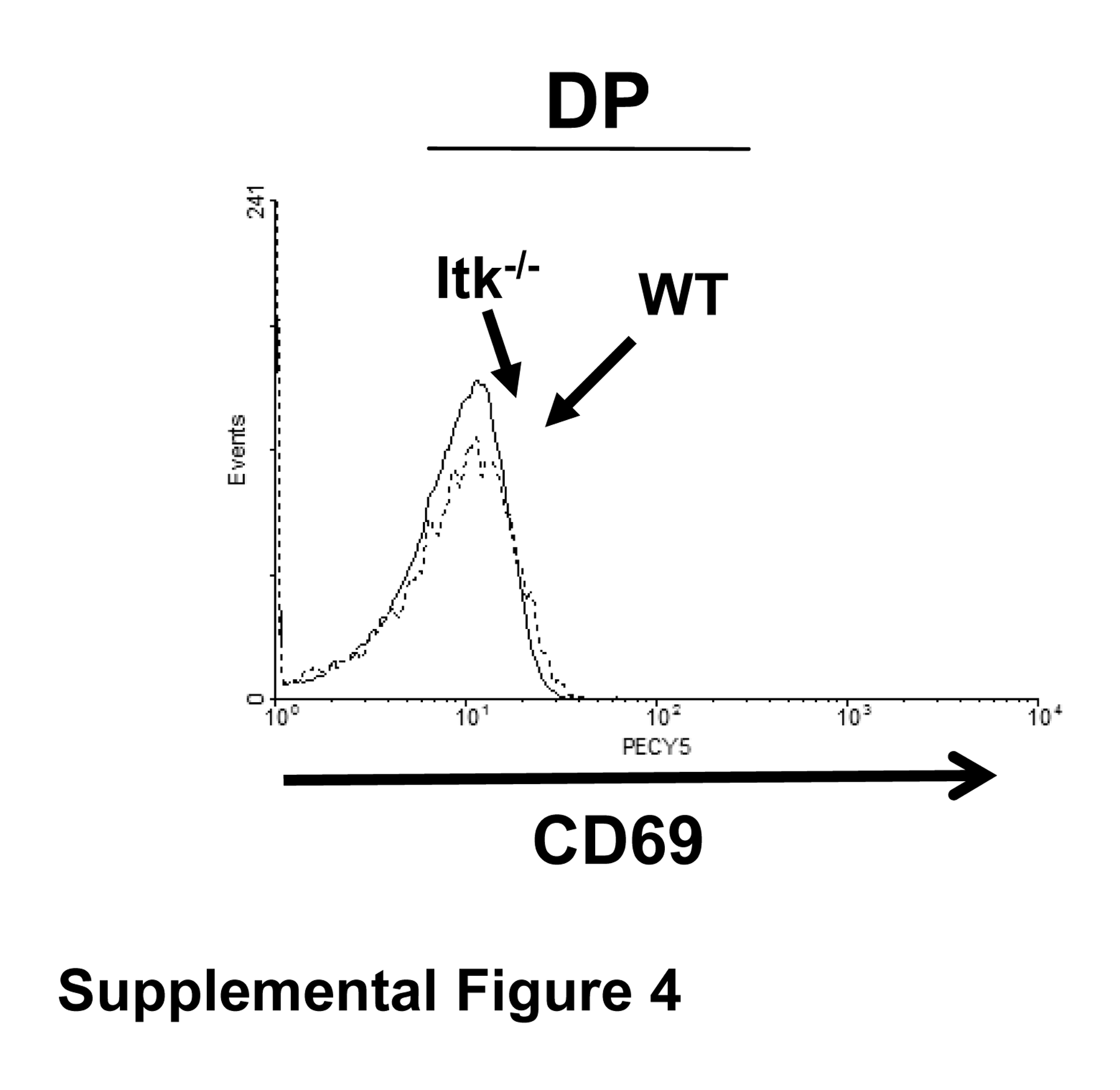

Supplement: Figure S4 — CD69 expression and gene expression profiles of naïve (conventional) CD4SP thymocytes in the OT-II transgenic system. Thymocytes from TCR transgenic OT-II and OT-II/Itk−/− were stained with antibodies against CD4, CD8α and CD69. Representative histograms of CD69 expression on gated CD4/CD8 DP thymocytes are shown for OT-II (solid line) and OT-II/Itk−/− (dashed line). (3.91 MB TIF) [file pone.0008891.s004.tif]
